# Upper body thermal images and associated clinical data from a pilot cohort study of COVID-19

**DOI:** 10.1186/s13104-024-06688-w

**Published:** 2024-01-19

**Authors:** Sofia Rojas-Zumbado, Jose-Gerardo Tamez-Peña, Andrea-Alejandra Trevino-Ferrer, Carlos-Andres Diaz-Garza, Meritxell Ledesma-Hernández, Alejandra-Celina Esparza-Sandoval, Rocio Ortiz-Lopez, Guillermo Torre-Amione, Servando Cardona-Huerta, Victor Trevino

**Affiliations:** 1 Tecnologico de Monterrey, Escuela de Medicina y Ciencias de la Salud, 64710 Monterrey, Nuevo León México; 2 Tecnologico de Monterrey, Hospital Zambrano Hellion, 66278 San Pedro Garza García, Nuevo León México

**Keywords:** Covid-19, Thermal imaging, Thermal videos, Respiratory disease, Demographic data

## Abstract

**Objectives:**

The data was collected for a cohort study to assess the capability of thermal videos in the detection of SARS-CoV-2. Using this data, a published study applied machine learning to analyze thermal image features for Covid-19 detection.

**Data description:**

The study recorded a set of measurements from 252 participants over 18 years of age requesting a SARS-CoV-2 PCR (polymerase chain reaction) test at the Hospital Zambrano-Hellion in Nuevo León, México. Data for PCR results, demographics, vital signs, food intake, activities and lifestyle factors, recently taken medications, respiratory and general symptoms, and a thermal video session where the volunteers performed a simple breath-hold in four different positions were collected. Vital signs recorded include axillary temperature, blood pressure, heart rate, and oxygen saturation. Each thermal video is split into 4 scenes, corresponding to front, back, left and right sides, and is available in MPEG-4 format to facilitate inclusion into pipelines for image processing. Raw JPEG images of the background between subjects are included to register variations in room temperatures.

## Objective

COVID-19 is a respiratory disease caused by the coronavirus SARS-CoV-2 [1]. The respiratory illness may cause acute respiratory distress syndrome (ARDS) characterized by pulmonary infiltrates and hypoxemia, where dry cough, fever, and fatigue are the main symptoms [2, 3].

Imaging methods such as computed tomography [4, 5] have been proposed as an alternative diagnostic tool for the SARS-CoV-2 coronavirus. Nevertheless, many of these methods may not be feasible for mass screening. CT uses ionizing radiation, requires unique installations and a complicated process that limits the number of possible tests per equipment, and the economic costs can be prohibitively high for screening a large population. Although thermal imaging is a cost-effective method used often in industry [6–8], to our knowledge, thermal imaging and specifically thermal videos have not been comprehensively investigated as an alternative diagnostic tool for COVID-19. Infrared videos can be useful for COVID-19 detection because SARS-CoV-2 infection in viremia stages is characterized by body temperature changes and breathing patterns [9]. Thus, video recording of body temperatures could theoretically be used as an alternative, cheap, and massive screening method for detection at an early stage of the disease.

The data was collected for use in a prospective study regarding the use of thermal data for Covid-19 detection, and was used in a publication titled “COVID-19 classification using thermal images” [10].

The database includes general patient and demographic information that could be of use in additional studies for associations in temperature patterns; for instance, assessing symptoms and physiological responses to medications or lifestyle factors.

## Data description

The study cohort comprises 252 volunteer subjects over 18 years old requesting a PCR test for SARS-CoV-2 at the TecSalud Zambrano-Hellion Hospital, Nuevo León, México in 2020. Individuals unable to hold a deep breath for at least 10 s were excluded. Table1 (Data file 5) shows the data fields collected. Vital signs were obtained using a Welch-Allyn 71WT monitor.

**Table 1:** Overview of data files/data sets

Label	Name of data file/data set	File types (file extension)	Data repository and identifier (DOI or accession number)
Data set 1	background_jpg	Image Files (.jpg)	PhysioNet (https://doi.org/10.13026/wfr2-5973) [11]
Data set 2	matlab_scripts	Matlab Scripts (.m)	PhysioNet (https://doi.org/10.13026/wfr2-5973) [11]
Data set 3	termal_avi_data	Video Files (.avi), Matlab Scripts (.m)	PhysioNet (https://doi.org/10.13026/wfr2-5973) [11]
Data set 4	termal_mpg_data	Video Files (.mp4)	PhysioNet (https://doi.org/10.13026/wfr2-5973) [11]
Data file 1	LICENSE	Text file (.txt)	PhysioNet (https://doi.org/10.13026/wfr2-5973) [11]
Data file 2	MEXICAN-DATA-ACCESS-FORM-ThermalCovid19	Word (.docx)	PhysioNet (https://doi.org/10.13026/wfr2-5973) [11]
Data file 3	MEXICAN-NON-DISCLOSURE-AGREEMENT	Word (.docx)	PhysioNet (https://doi.org/10.13026/wfr2-5973) [11]
Data file 4	SHA256SUMS.txt	Text file (.txt)	PhysioNet (https://doi.org/10.13026/wfr2-5973) [11]
Datafile 5	subject_description	Excel (.csv)	PhysioNet (https://doi.org/10.13026/wfr2-5973) [11]
Datafile 6	survey_template_datos_termico	PDF (.pdf)	PhysioNet (https://doi.org/10.13026/wfr2-5973) [11]

### Thermal image acquisition

Thermal videos were acquired at five frames per second, using a TI-128 Digital Thermal Imaging Camera from Omega Engineering Inc. (Norwalk, CT, USA). The camera was connected via USB to a computer running a Microsoft Windows operating system, and drivers and the Omega TI Analyzer version 4.1.8.6875 acquisition software were obtained and installed as per provider instructions.

Before recording, glasses, masks, caps, and t-shirts were removed. Undergarments and jewelry were not removed. Participants were recorded with raised arms, taking a breath, holding for 10 s, and again with their arms down. They then turned clockwise 90 degrees 3 times to repeat the process from the left, back, and right sides. For de-identification reasons, left and right side and raw videos were removed from the repository. Nevertheless, this information can be provided upon request. Background images were taken before or after each procedure to record variations in room temperatures.

### Image pre-processing

Raw background snapshots are included in this repository without any processing. In addition, we carefully split each video into four scenes corresponding to the front, left, back, and right sides and converted them to MPEG-4 format to facilitate their inclusion into pipelines for the image processing scientific community. For de-identification reasons, left and right side and raw videos were removed from the repository. Nevertheless, this information can be available upon request.

### COVID-19 PCR diagnosis

Nasopharyngeal swabs were taken by qualified personnel and deposited in a vital transport medium following standard procedures. The PCR testing was performed by the Clinical Laboratory from the San José Hospital and included TaqMan 2019-nCoV Assay Kit v1 & v2 from ThermoFisher. The PCR results yielded 59 PCR positives, 192 PCR negatives, and 1 not reported.

### Generating position files

The average duration of each video is 81 s (standard deviation of 19). Each thermal video was inspected to determine the interval for body positions. Each IRS video segment was then converted into a standardized black-and-white MP4 video whose gray levels were standardized between 62 and 102-degrees Fahrenheit. Four additional files were generated for each body position. The mean and standard deviation for the duration for each scene are 13.7 (3.5), 12.5 (3.4), 12.4 (3.5), and 12.1 (3.2) seconds for the *front, left, back,* and *right* respectively. Around 30 s of changing positions were manually removed, facilitating use, and standardizing future comparisons.

### Corrections

We noted variations in focus, thermal miss-calibration, frame rate, and camera orientation. For focus correction, we post-process each video frame and apply a deblurring operation. For frame rate, video clips with a frame rate higher than five frames per second were downsampled to five frames per second. Thermal variations were accounted for by monitoring the background temperature and body temperature for each frame. We then applied a linear transformation on each frame to enforce that all frames showed the same background and body temperature. Camera orientation was standardized. The final corrected video was stored in AVI format.

## Limitations

While care was taken to minimize artifacts, some subjects were nevertheless out of focus or moved during the acquisition period, and the camera error is expected to have some degree of interference in the results. Specifically, the measurement accuracy of the camera corresponds to ± 2 ℃ or ± 2% at environmental temperatures 10 ℃ to 35 ℃, while among the participants the temperature variance during the video capture was < 1.75 ℃ and 1.4 ℃ for negative and positive participants, respectively, with the median around 0.55 ℃ in both cases. Because this observational study has a small sample size, conclusions drawn from the data may be implemented as part of data exploration and proof-of-concept, and validated with a larger database for generalization. Final considerations for the use of this data include an imbalance between male and female subjects recorded, and inter-subject variations such as facial hair and jewelry.

## Data Availability

The data described in this Data note can be freely and openly accessed on PhysioNet under https://doi.org/10.13026/wfr2-5973. Access to the data record will require registration with PhysioNet and compliance with a data use agreement. Please see Table 1 and reference [11] for details and links to the data.

## Abbreviations

WHO: World Health Organization
PCR: Polymerase chain reaction
CT: Computed tomography
